# Temporal Validation of Chiang Mai University Intussusception Failed Reduction Score (CMUI)

**DOI:** 10.3390/ijerph19095289

**Published:** 2022-04-26

**Authors:** Jiraporn Khorana, Chanathip Sayuen, Sutinee Chanaturakarnnon, Butsarin Nate-anong, Jesda Singhavejsakul, Kanokkan Tepmalai, Sireekarn Chantakhow, Wilai Sathavornvichit

**Affiliations:** 1 Division of Pediatric Surgery, Department of Surgery, Faculty of Medicine, Chiang Mai University, Chiang Mai 50200, Thailand; abtsportblack@gmail.com (C.S.); sutinee_ch@cmu.ac.th (S.C.); butsarin_n@cmu.ac.th (B.N.-a.); jesda.s@cmu.ac.th (J.S.); kanokkan.t@cmu.ac.th (K.T.); sireekarn.chan@cmu.ac.th (S.C.); 2 Center of Clinical Epidemiology and Clinical Statistic, Faculty of Medicine, Chiang Mai University, Chiang Mai 50200, Thailand; 3 Clinical Surgical Research Center, Department of Surgery, Faculty of Medicine, Chiang Mai University, Chiang Mai 50200, Thailand; 4 Pediatric Nursing Section, Faculty of Medicine, Chiang Mai University, Chiang Mai 50200, Thailand; wilai.sa@cmu.ac.th

**Keywords:** intussusception, nonoperative reduction, predictor score, pediatric

## Abstract

This study aimed to validate the “Chiang Mai University Intussusception Failed Score (CMUI)” for intussusception non-operative reduction. Both a 2-year retrospective and a 5-year prospective consecutive review of patients with intussusception were conducted. Data were collected from January 2013 to December 2020. Related retrospective data of a developmental set from two centers from January 2006 to December 2012 were used. Ten prespecified prognostic factors for failed reduction were collected and from these a predictive score was calculated. The actual results of non-operative reduction were collected and set as a reference standard. Altogether, 195 episodes of intussusception were found. Twenty-two patients were excluded due to contraindications; therefore, a total of 173 episodes were included in the validation dataset. The development data set comprised 170 episodes. We found that no statistical significance was found from comparing the areas under the ROC of two datasets (*p*-value = 0.31), while specificity of the validation set was 93.8% (88.1–97.3). This temporal validation showed a high specificity and a high affinity for prediction of failed reduction as the development dataset despite being in an era of a higher successful reduction rate. The intensive reduction protocols might be introduced among patients with high-risk scores.

## 1. Introduction

Intussusception is a common surgical emergency and a frequent cause of bowel obstruction and lower gastrointestinal bleeding among infants and children with an incidence of between 1 and 4 per 2000 infants and children, respectively [1,2,3]. Delay in diagnosis and treatment could lead to serious complications such as bowel perforation, bowel ischemia, and peritonitis. Intussusception could be diagnosed according to the clinical case definition proposed by the Brighton Collaboration Intussusception Working Group and confirmed by ultrasound [4,5]. Currently, the treatment modalities for intussusception consist of operative and non-operative treatment. The non-operative treatment is the first step if no contraindications present, contraindications being hemodynamic instability despite adequate resuscitation, peritonitis, and abdominal X-ray signs of pneumoperitoneum. The success rate of non-operative reduction in related reports varied from 46 to 94% [6]. The success rate is currently increasing due to the improved reduction technique and wider knowledge about the disease resulting in early consultation. Surgical treatment is preserved when nonsurgical treatment is contraindicated or has failed. However, some patients were operated on immediately because of many limitations for nonoperative reduction such as referral problems, and a lack of availability of facilities in small hospital centers [7,8]. The parents need to be advised regarding the option of nonoperative reduction if possible. Referral of the cases to centers with available facilities for nonoperative reduction should be considered when chance of failed nonoperative reduction exists at smaller centers.

The techniques of intussusception reduction have improved and developed in many aspects. Our first study about intussusception showed that pneumatic reduction showed a 1.48 times higher success rate than hydrostatic [9]. Sedation was also shown to be one of the keys to improving the success rate. A recent study reported a higher success rate with general anesthesia rather than sedation [10]. Therefore, predicting those patients with a high chance of failed reduction may aid the decision making of the care team regarding to the technique used for reduction.

Our second series of studies in intussusception showed ten prognostic indicators for failure of non-operative reduction [11]. The clinical prediction rules for failed non-operative reduction which are subsequently referred to as “Chiang Mai University Intussusception (CMUI) Failed Score” was established in our third series [12]. The prognostic factors for failed reduction were bodyweight less than 12 kg, duration of symptoms more than 48 h, vomiting, rectal bleeding, abdominal distension, temperature more than 37.8 °C, palpable mass, location of mass on the left side, poor prognosis signs from ultrasound and method of nonoperative reduction, i.e., hydrostatic. The assigned scores for each parameter were transformed from the coefficient of the regression model of the statistically significant factors associated with failed nonoperative reduction detailed in our third series [12]. 

This study is the fourth in a cluster of study series regarding intussusception in a tertiary center. This study aimed to evaluate the application of the scoring system constructed from the third study in the different settings. This was a temporal validation and the validation across the time of scoring guidelines in clinical prediction rules for failed nonoperative reduction known as CMUI. 

## 2. Materials and Methods

This validation study of the clinical prediction rule was described using transparent reporting of a multivariable prediction model for individual prognosis or diagnosis (TRIPOD).

### 2.1. Source of Data

The validation data set consisted of data from the retrospective cohort study between January 2013 and December 2015 and prospective consecutive cohort study between January 2016 and December 2020. The study was approved by the Ethics Committee of Chiang Mai University (CMU) Hospital- STUDY CODE: SUR-2559-03895/Research ID 3895. The patient informed consent was waived in the retrospective part and prospective non-interventional part was verbally consented to by the parents or guardian of the participants. The developmental data set was retrospectively collected in two centers, CMU (northern Thailand) Hospital and Siriraj Hospital (central Thailand) between January 2006 and December 2012.

### 2.2. Participants

In the validation set, all intussusception patients (ICD-10 code K56.1) visiting CMU Hospital in the specified period, mentioned above, were collected. The inclusion criterion was patients aged 0 to 15 years. The exclusion criteria included patients who had contraindication for non-operative reduction, spontaneous reduction before treatment, and when there had been no attempt at non-operative reduction. 

### 2.3. Non-Operative Reduction

In CMU Hospital, all patients with intussusception received pneumatic reduction performed by a radiologist and pediatric surgeon under fluoroscopic guidance. These procedures were performed among well-hydrated children. Sedation drugs were administered according to hospital sedation guidelines by a pediatric surgeon, pediatrician, or anesthetist. A Foley catheter was inserted through the anus and the buttocks were taped to prevent air leakage. Air pressure from 80 to 120 mmHg was used in each case. The standard techniques of reduction comprised three repeated attempts of three minutes each with no more than three attempts. The success of reduction was determined by the disappearance of intussusception and the visualization of air from the cecum to the ileum through the ileocecal valve under fluoroscopic view, and absence of intussusception soft tissue density after reduction by fluoroscopic view and post reduction ultrasound examination. 

### 2.4. Predictors

The data were obtained by chart review and electronic databases in the retrospective data collection then collected and recorded on an electronic program in the prospective part. Ten predictors included bodyweight, duration of symptoms, vomiting, rectal bleeding, abdominal distension, temperature, palpable mass, location of mass, poor prognosis signs from ultrasound, and method of nonoperative reduction. The signs of a poor prognosis from the ultrasound were counted if one of the signs already mentioned was present, specifically thick peripheral hypoechoic rim, free intraperitoneum fluid, fluid trapped within the intussusception, enlarged lymph node in the intussusception, pathologic leading point, or absence of blood flow in the intussusception. The methods of nonoperative reduction carried out were pneumatic reduction and hydrostatic reduction. In the validation set, the method of reduction was always pneumatic reduction in line with hospital policy and the results of the related study. Laboratory investigation data and the results of plain abdominal x-rays were also collected.

All episodes of intussusception were collected. The CMUI scores ranging from 0 to 16 were assigned to each predictor (Table 1) [12]. A total score of 0 to 11 was classified in the low chance for failure reduction group, and a total score of 12 to 16 was classified in the high chance for failure reduction group. The point of prediction was the time of the patient visit and diagnosis of intussusception by ultrasound. The assessor and care team obtained the score before the reduction process and the result of reduction was naturally blind in the prospective part of the collection. The electronic calculator of the prediction score was placed on “https://w1.med.cmu.ac.th/surgery/personnel/pedsurgerycmu/#1648632882495-6c8cbc3e-1729” (accessed on 12 February 2022).

### 2.5. Outcome

Results of the nonoperative reductions were collected as the outcome of the study. The patients were divided into two groups, which were failed and successful reduction. 

### 2.6. Sample Size

The sample size was calculated based on the test of two independent proportions. From the developmental data set, the low-risk score group had a failed reduction rate of 41% and the high-risk score group had a failed reduction of 94% [12]. With a significance level (α) of 0.05 and a power (β) of 0.80, and the ratio of success to failed reduction of 1.3 the approximate total sample size was 17 in the success group and 13 in the failed group. In this validation study, the total failed events of 45 out of a total number of 173 were included.

### 2.7. Missing Data

Data were missing in the developmental set. The previous construction of the CMUI score used complete case analysis. However, in this study we used multiple imputation with chained equation (MICE) for imputation of the missing data of the model including parameters, i.e., left sided location of mass and ultrasound showed poor prognosis signs. (Missing at 3, and 15 out of 170 participants, respectively.) Missing parameters that were not included in the model were not imputed and shown as complete case analysis data. 

### 2.8. Statistical Analysis

The statistical analysis was performed using commercial statistical software (STATA 16.0; StataCorp LP, College Station, TX, USA). Comparisons between the developmental and validation data sets were carried out. The descriptive data were reported as number and percentage for categorical data. Mean and standard deviation or median and interquartile range were reported for continuous data depending on data distribution. The univariable analysis was carried out using Fisher’s exact test for categorical data and a Student’s *t*-test or Mann–Whitney U test for continuous data. The multivariable analysis was performed using an exponential risk regression model clustering the data in age groups of three years with ten predefined predictors from the developmental model. The statistical significance level was set as two-tailed with a *p*-value < 0.05. 

The internal validation of the developmental dataset and the external validation of the validation dataset were preformed using the bootstrapping procedure with 1000 replicates reported by model optimism, calibration in the large (CITL), and shrinkage factor.

The validation data were compared using the developmental data by areas under the receiver operating characteristic curves (ROC). A comparison of the probability of failed reduction by development and validation datasets is shown in a bar chart with error bars. The predictive ability of the scoring system of both datasets was graphically compared by the probability or risk curves. Hosmer–Lemeshow goodness of fit statistics and calibration plot comparing the agreement of observed and expected score values were also presented.

## 3. Results

**Figure 1:**
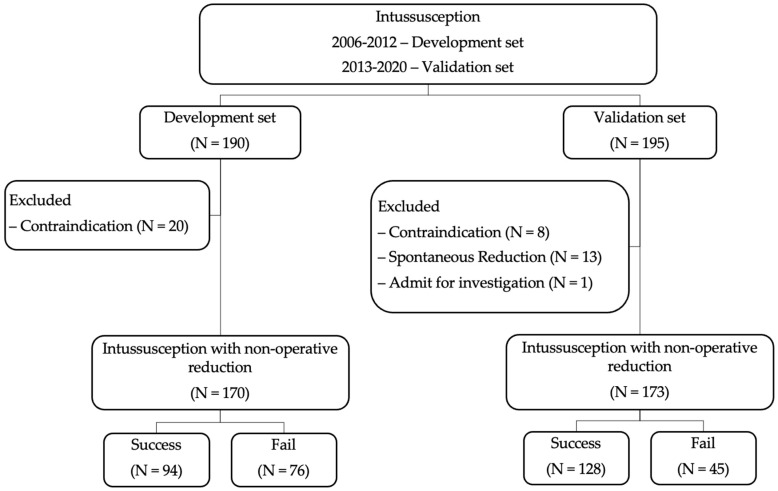
Study flow diagram.

In this validation dataset, a total of 195 episodes of intussusception were identified. Twenty-two patients were excluded due to contraindication for nonoperative reduction, spontaneous reduction, and admission for investigation. One hundred and seventy-three episodes were included in the validation set of this study (Figure 1). 

The development dataset totaled 190 episodes of intussusception. After exclusion of 20 episodes of contraindication, 170 episodes were included in the related study [12]. One hundred and fifty-four cases were finally included in the previous study as complete case analysis basis. In this study, we imputed the 16 episodes of two missing predictors accounting for a total of 170 episodes in the final analysis. 

The comparative baseline characteristics among the development and validation datasets are shown in Table 2. These showed the comparative characteristics of the patient between era leading to the validation of the scoring system across the time and population. The parameters which were found to be significantly different between the two datasets were location of the mass, plain abdominal film showing small bowel obstruction, age of presentation, bodyweight, chloride, and carbon dioxide levels.

The comparative ten CMUI predictors, scores, and results of reduction among the development and validation datasets are shown in Table 3 and Table 4. The parameters which significantly differ between the two datasets were presence of ultrasound poor prognostic signs and method of reduction. The validation set showed a higher percentage of the ultrasound poor prognostic signs and the method of reduction used in the latter era were only the pneumatic reduction as stated before. The success rate between the two datasets was significantly difference (55.3% vs. 74%, *p*-value < 0.001). In the validation dataset, 149 episodes (86.1%) were predicted as the low chance for failure reduction group according to CMUI predictor scores. However, 128 episodes (74.0%) were successful nonoperative (pneumatic) reductions. Twenty-nine (16.8%) patients having a low chance for failed reduction had failed result and eight (4.6%) patients having a high chance of failed reduction had success result. These showed the misclassification percentage of the failed score was lower in the high-risk group. As the purpose of the score construction was to encourage the reduction and provide the affinity to detect the high-risk group, we preferred the lower percentage of misclassification in the high-risk group. 

**Figure 2:**
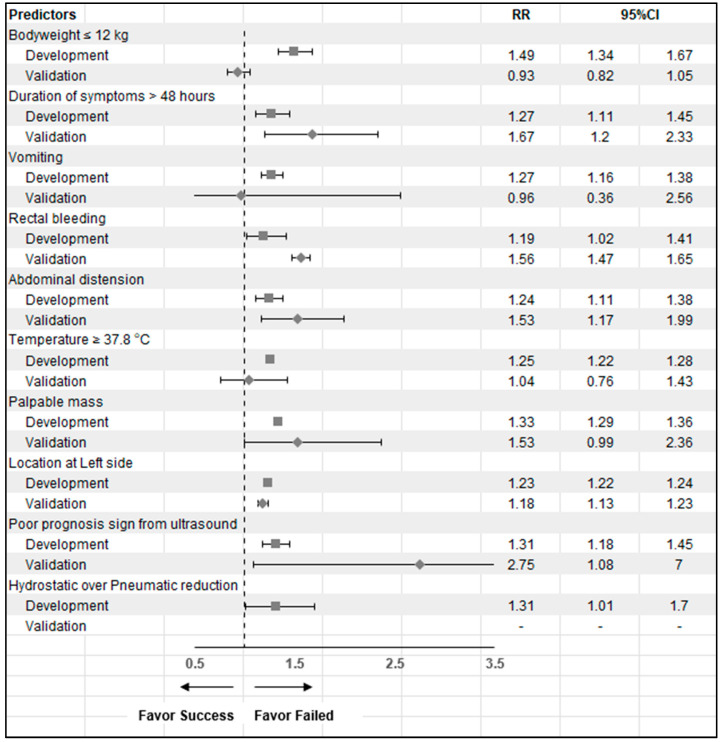
Risk ratio plots of the ten-predictors of CMUI in the development and validation sets.

Risk ratio (RR) of the ten CMUI predictors compared between the two datasets are shown in Figure 2. The risk ratio plot showed almost the same direction of prediction in ten parameters except for bodyweight and the presentation of vomiting (RR < 1) in the validation dataset but without significance. The most potent predictive factor for failed reduction in the validation set was poor prognosis signs from ultrasound (RR = 2.75 (1.08–7)).

In the validation dataset, the sensitivity, specificity, likelihood ratio, and predictive value of CMUI at a cut point of high risk of failed reduction of more than 11 points; ≥12 are shown in Table 5. The details of each cut off point re shown in Table 6. The concern of this scoring system was to achieve a high specificity because if no contraindication for reduction exists, we promote receiving the nonoperative reduction of every case. The prediction for failed reduction supported the patient preoperative management protocol such as intensive intravenous fluid resuscitation or the depth of sedation during reduction. In our study, the cutoff point of 12 showed the specificity more than 90% and was chosen. 

One hundred and forty-nine episodes of intussusception had low risk for failed reduction by CMUI score (Table 4). One hundred and twenty-eight episodes had successful reduction. Of these, 120 of low risk out of 128 of true successful episodes did not require surgery. This true negative proportion resulted in a specificity of 93.8%.

**Figure 3:**
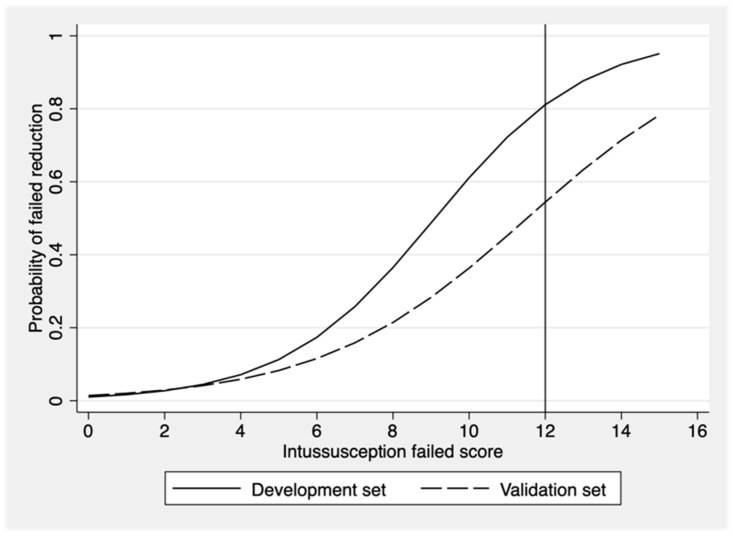
Score-predicted probability of failure of nonoperative reduction of intussusceptions between the developmental set and the validation sets with a cut point score of 12.

**Figure 4:**
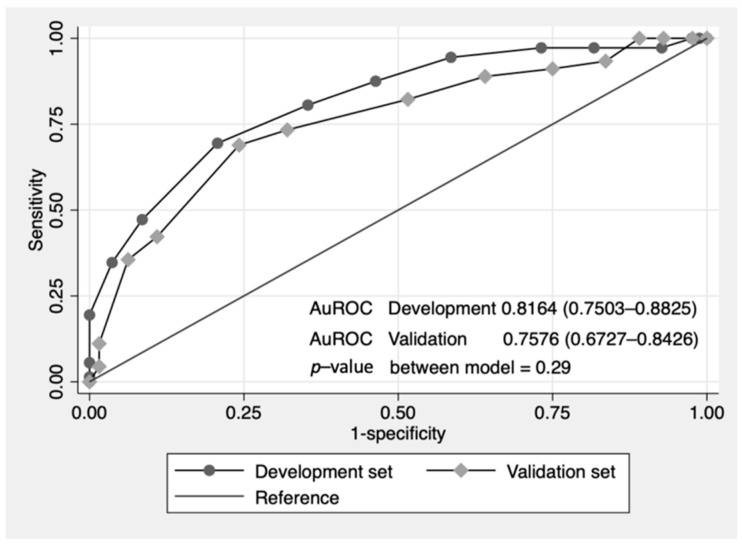
Comparative ROC curve of failure of nonoperative reduction of intussusceptions between the developmental set and the validation set predicted by risk scoring scheme (curved line) and a 50% chance prediction (diagonal line).

Score-predicted probability of failure of nonoperative reduction between the developmental set and the validation set with the cutoff point score of 12 is shown as a risk curve in Figure 3. The cutoff point of 12 showed the probability of failed reduction at more than 50% in both development and validation datasets. The ROC curve of failed nonoperative reduction predicted by the risk scoring scheme of CMUI was performed. The area under the ROC curve that determined the prediction ability of the score model was 81.6% in the development group and 75.8% in the validation group as shown in Figure 4. No statistically significant difference was found between the ROC of the two datasets (*p*-value = 0.29). The validation dataset showed acceptable predictive ability. 

**Figure 5:**
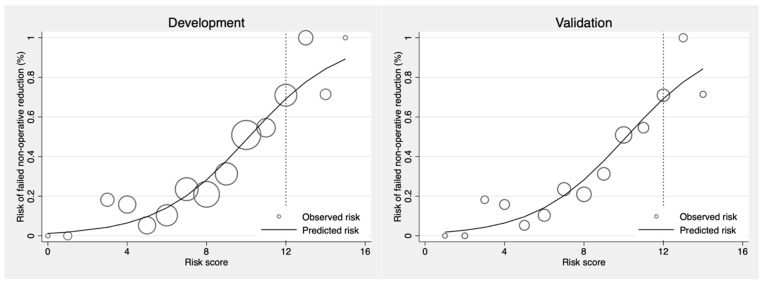
Calibration plots of the CMUI score in the development and validation sets. The dash lines at the cutoff point of 12 categorized patients with intussusception into low-risk and high-risk groups of failed non-operative reduction.

**Figure 6:**
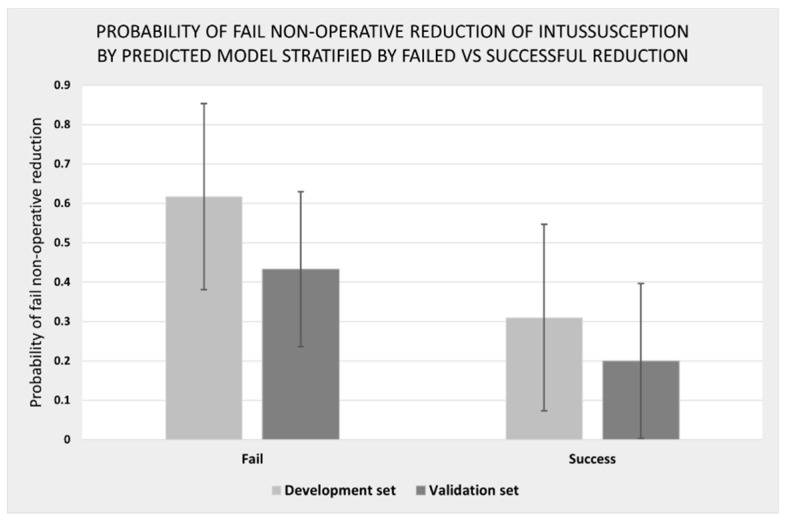
Probability of fail of non-operative reduction of intussusception by predicted model stratified by failed vs. successful reduction in the development and the validation sets.

The calibration plots of the CMUI score in the development and validation set are compared in Figure 5. The agreement between the score predicted probabilities and the observed proportion of failed reduction was acceptable for which the observed events, shown as circles, almost lay on the predictive line. Probability of failed of non-operative reduction by the predicted model stratified by failed vs. successful reduction in the development and the validation sets is shown in Figure 6. This showed the discriminative valued failed the probability test among the failed and successful groups in both development and validation datasets. 

In the development dataset, the Hosmer–Lemeshow goodness of fit statistics was carried out for the ten parameters model and the score model without finding statistical significance found (*p*-value = 0.629 and 0.579, respectively). The CMUI development model was a good for predictor of failed reduction. Internal validation and external validation performance were performed using the bootstrapping method with 1000 replications. Internal validation of the developmental model showed an apparent area under the ROC of 0.84 ± 0.03 with model optimism at 0.04 (range from −0.07–0.13). C-statistic, CITL, and shrinkage factors indicated good calibration performance in both internal and external validation as shown in Table 7. 

In the validation dataset, 45 episodes of intussusception were failed nonoperative reduction, and surgery was performed immediately after adequate resuscitation. Of these patients, 17 (38%) patients required manual reduction, 12 (27%) patients required small bowel resection with anastomosis due to bowel ischemia, 10 (22%) patients had pathologic leading points, and 6 (13%) patients experienced spontaneous reduction. Pathologic leading points involved six patients with Meckel’s diverticulum, and one patient each with duplication cyst, polyp, acute appendicitis, and lymphoma. Thirteen out of 173 episodes were recurrent episodes accounting for 7.5%.

**Figure 7:**
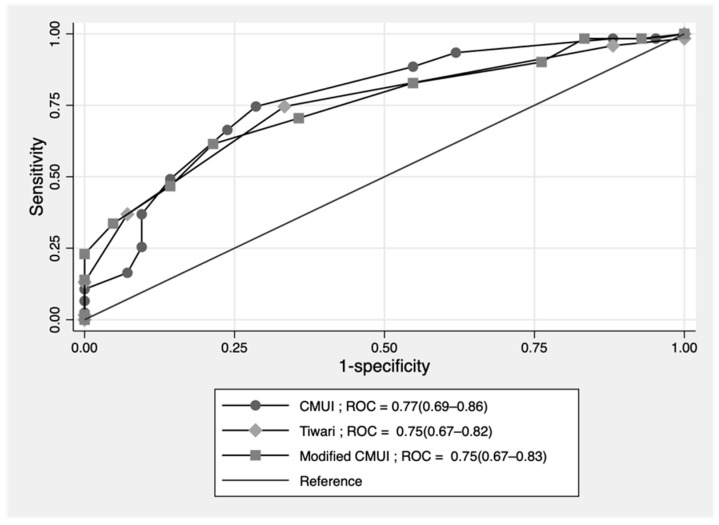
Comparative ROC curve of failure of nonoperative reduction of intussusceptions among 3 score models.

The comparative score validation with the recently published clinical scoring system within 5 years was performed. In 2019, a modified CMUI model published by Boonsanit, K. et al. proposed 10 parameters with systemic scoring showing an area under the ROC of 74.78% (66.90–82.65%) [13]. In 2020, a clinical score model constructed by Tiwari, C. et al. with six parameters showed an area under the ROC of 74.63% (67.00–82.26%) [14]. These two models were created using 164 episodes of our validation dataset with no missing parameters. The CMUI model showed an area under the ROC of 77.10% (68.53–85.67%), and the comparative ROC curve among the three models is shown in Figure 7.

## 4. Discussion

This study was the fourth in a series of studies on intussusception conducted in our institution which was an external validation of the CMUI in terms of temporal validation in different time periods and in the subdomains of the related developmental series which was constructed from the university hospitals in two regions of Thailand. All patients had pneumatic reduction under fluoroscopic guidance by a radiologist. This method is now solely used as in the first series of the study of pneumatic reduction revealing a higher success rate than hydrostatic reduction [9]. In this validation study, the success rate of nonoperative reduction was higher than that in the related study (74% vs. 55%). This validation study could also show the performance of the score with a different prevalence in the success rate. 

The discriminative performance of CMUI in the development and validation dataset was 81.24% and 75.76%, respectively (area under ROC). Although, a decrease was observed in the validation set, the performance was still acceptable. This score had been constructed from the North and Central University Hospital of Thailand and had been validated in the university hospital in southern of Thailand in a fully independent validation [13]. In that study, 73% area under ROC was obtained with the original CMUI. In the modified CMUI, the investigation data were added, i.e., sodium level and different cutoff point of bodyweight to replace the method of reduction which increase the area under ROC to 76%. To generalize the score, our study still used the method of reduction as a predictor. The actual point of prediction using the CMUI was made at the time of the patient visit and diagnosis of intussusception by ultrasound. The result of the investigation might be unavailable. Therefore, we still used the ten predictors model to predict failed reduction.

The other clinical scoring system was used by Tiwari, C. et al. with six parameters, i.e., age, duration of symptoms, abdominal distension, abdominal mass, and currant jelly stool. This scoring system was assigned by the results of the reduction [14]. CMUI score assignment was performed by transforming the regression coefficient of the regression analysis. Among the three score models, CMUI had the highest area under ROC (77% vs. 75% vs. 75%).

In 2021, a meta-analysis was conducted by Kim, P.H. et al. reporting the similar predictors of failed reduction as our study [15]. Some differed such as duration of symptom cut-off point in their study was 24 h compared with 48 h in our study [16]. The longer duration was associated with the compromised bowel resulting in failed reduction. Other interesting parameters were age and bodyweight. Most studies proposed age as the predictor as well as in this meta-analysis. We used bodyweight instead of age because some of the patient did not have the actual bodyweight at the specific age, and size of the intestinal lumen depended on the body size, Smaller luminal size might have been associate with primary intussusception from the hypertrophied of Payer’s patch. The other predictors were quite the same, i.e., vomiting, rectal bleeding, fever, left sided intussusception, and poor ultrasonographic sign, which was associated with the greater severity of the disease [16,17]. In 2018, Gondek, A.S. et al. designed a mathematical model using three parameters, i.e., onset of symptoms, free peritoneal fluid, and intussusception location resulting in an area under ROC of 67.3% [18]. Another study by Ajao, A.E. et al. in 2020 predicted that fever, abdominal pain, abdominal distension, rectal mass, age less than 12 months, heart rate more than 145 times per minute and duration of symptoms more than 2 days were associated with bowel resection [19]. In our study, the CMUI systematic scoring was validated by applying it across the time, domain, and the difference of the success rates of reduction. The level of performance was still acceptable.

One hundred and forty-nine episodes were predicted to have a low chance of failed reduction and 24 to have a high chance failed reduction. Altogether, 45 failed reduction episodes with 29 episodes were predicted to be low chance. Thus, this scoring system exhibited a low sensitivity because we were advocating reduction if no contraindication. Our selected cut point was set for high specificity. Twenty-nine episodes showed a score less than 12, and low chance for failure, but actually failed reduction occurred. In all, 12 difficult manual reduction cases, 4 bowel ischemia, 9 pathologic leading points, and 4 intraoperative spontaneous reductions were observed. These findings led to an understanding that the low chance group with intraoperative spontaneous reduction could be improved using the reduction technique, and the remainder could not be avoided but should be suspected to require surgical correction. A successful nonoperative reduction could be improved by many factors such as adequate sedation [20], dehydration status, continuity of pressure application, and experience of the surgeon or radiologist who performed the procedure. Various protocols in intussusception reduction were observed across the institute. A more aggressive protocol may be introduced among those patients exhibiting high-risk score, and this may include deep sedation, adequate decompression, and hydration and prompt family advice and counselling. However, non-operative reduction should be attempted even in high-risk groups unless the presence of contraindications is detected.

Limitations were encountered this study. Firstly, the validation dataset was not entirely prospective. Of this 8-year external validation study, the first 3 years were retrospective and the latter 5 years were prospective. However, in the retrospective period the systematic data collection was well planned after score development resulting in no missing data of the predictors. Secondly, only one single method of reduction in the validation dataset. All patients underwent pneumatic reduction. Although this predictor was unused, we still maintained the method of reduction as a predictor because of the generalizability of CMUI to other institutions with both or any of modalities which could have been an important predictor. This might be one of the reasons for the slight decrease in the area under ROC in the validation setting.

We recommend CMUI to predict failure of nonoperative reduction. The predictor scores have a high specificity that were effectively used to predict the results of nonoperative reduction and forecast the prognosis of failed nonoperative reduction among patients with intussusception patients.

## 5. Conclusions

This temporal validation showed high specificity and a likelihood ratio of positive. The validation dataset also showed a high affinity for prediction, as the development dataset, despite being in the era of a higher successful reduction rate. The remote hospitals without nonoperative options were encouraged to refer the patients to the more specialist centers and parental concern was successfully addressed by the use of this scoring system. More intensive reduction protocols might be introduced among patients with high-risk scores.

## Figures and Tables

**Table 1:** Ten predictors of CMUI with score assignment.

Predictor	Score
Bodyweight ≤ 12 kg	2
Duration of symptoms > 48 h	1
Vomiting	2
Rectal bleeding	2
Abdominal distension	2
Temperature ≥ 37.8 °C	2
Palpable mass	1
Location on the Left side	2
Poor prognosis signs from ultrasound	1
Method of reduction	
Pneumatic reduction	0
Hydrostatic reduction	1

**Table 2:** Baseline characteristics between development set (N = 170) (Showing missing data) and validation set (N = 173) of intussusception patients.

**Baseline Characteristic**	**Development Set** **(N = 170)**			**Validation Set** **(N = 173)**		***p* Value**
	**Total**	**n**	**%**	**n**	**%**	
Male	170	114	67.1	99	57.2	0.075
Location of Mass	167					
Right Lower Quadrant		15	9.0	20	11.6	0.037
Right Upper Quadrant		97	58.1	113	65.3	
Left Upper Quadrant		31	18.6	27	15.6	
Left Lower Quadrant		23	13.8	9	5.2	
Rectum		1	0.6	4	2.3	
Plain Abdominal film showed small bowel obstruction	160	107	66.9	71	41.0	<0.001
**Baseline Characteristic**	**Development Set** **(N = 170)**			**Validation Set** **(N = 173)**		***p* Value**
	**Total**	**Mean**	**SD**	**Mean**	**SD**	
Age (month) *	170	9.0	7.0–16.0	13.0	8.0–25.0	<0.001
Weight (kilogram)	170	9.5	3.3	10.3	4.0	0.031
Duration of Symptoms (hour) *	170	24.0	20.0–48.0	24.0	14.0–48.0	0.083
Temperature (°C)	170	37.3	0.7	37.3	0.6	0.900
White blood cell count (cells/mm^3^) *	163	12,000.0	9030.0–15,800.0	11,200.0	8930.0–14,600.0	0.270
Neutrophil count (%)	163	56.4	16.6	54.8	17.0	0.380
Sodium (mEq/L)	161	137.0	4.1	136.3	3.5	0.120
Potassium (mEq/L)	161	4.2	3.2	3.9	0.6	0.290
Chloride (mEq/L)	161	103.2	5.4	100.5	4.6	<0.001
Carbon dioxide (mEq/L)	161	19.4	3.6	17.8	2.7	<0.001

Notes: * Reported as Median and Interquartile range. Abbreviations: SD, Standard Deviation; mm^3^, cubic millimeter; mEq/L, milliequivalent per liter.

**Table 3:** Comparison of ten score variables between development set (N = 170) (imputed data) and validation set (N = 173) of intussusception patients.

Variables	Development Set (N = 170)		Validation Set (N = 173)		*p* Value
	n	%	n	%	
Weight ≤ 12 kg	147	86.5	143	82.7	0.370
Duration of Symptoms > 48 h	30	17.6	31	17.9	1.000
Vomiting	147	86.5	149	86.1	1.000
Rectal Bleeding	119	70.0	107	61.8	0.140
Abdominal Distension	78	45.9	91	52.6	0.240
Temperature > 37.8 °C	37	21.8	28	16.2	0.220
Palpable mass	113	66.5	101	58.4	0.150
Mass Located on Left Side	56	32.9	40	23.1	0.054
Presence of Ultrasound Poor Prognostic signs	81	47.7	133	76.9	<0.001
Method of Reduction					
Hydrostatic Reduction	59	34.7	0	0.0	<0.001
Pneumatic Reduction	111	65.3	166	100	

**Table 4:** Comparison of the total CMUI score and the result of reduction between development set (N = 170) (imputed data) and validation set (N = 173) of intussusception patients.

**Parameters**	**Development Set** **(N = 170)**		**Validation Set** **(N = 173)**		***p* Value**
	**Mean**	**SD**	**Mean**	**SD**	
Total score	8.5	2.9	8.0	3.0	0.084
**Parameters**	**Development Set** **(N = 170)**		**Validation Set** **(N = 173)**		***p* Value**
	**n**	**%**	**n**	**%**	
Intussusception failed score					
Low (0–11)	142	83.5	149	86.1	0.302
High (12–16)	28	16.5	24	13.9	
Result of reduction					
Success	94	55.3	128	74.0	<0.001
Fail	76	44.7	45	26.0	

**Table 5:** Indices of the validation of clinical prediction rule for failed reduction of intussusception; CMUI (cut point of high risk of failed reduction of more than 11 points; ≥12).

Indices	%	95% Confidence Interval
Sensitivity	35.6	21.9–51.2
Specificity	93.8	88.1–97.3
Likelihood ratio positive	5.7	2.6–12.4
Likelihood ratio negative	0.7	0.6–0.9
Positive predictive value	66.7	44.7–84.4
Negative predictive value	80.5	73.3–86.6

**Table 6:** Indices of the validation of clinical prediction rule for failed reduction of intussusception against various CMUI score cut points.

Cut Point	Sensitivity (%)	Specificity (%)	LR+	LR−
1	100.0	0.0	1.0	-
2	100.0	2.3	1.0	0.0
3	100.0	7.0	1.1	0.0
4	100.0	10.9	1.1	0.0
5	93.3	16.4	1.1	0.4
6	91.1	25.0	1.2	0.4
7	88.9	35.9	1.4	0.3
8	82.2	48.4	1.6	0.4
9	73.3	68.0	2.3	0.4
10	68.9	75.8	2.8	0.4
11	42.2	89.1	3.9	0.6
12	35.6	93.8	5.7	0.7
13	11.1	98.4	7.1	0.9
14	4.4	98.4	2.8	1.0
15	0.0	100.0	1.0	-

Abbreviations: LR+, likelihood ratio of positive; LR−, likelihood ratio of negative.

**Table 7:** Internal and external validation model calibration parameters with 1000 replications bootstrapping method (95% confidence intervals).

Parameters	C-Statistic	Calibration in the Large (CITL)	Shrinkage Factor
Apparent performance	0.83 (0.76–0.89)	0.00 (−0.36–0.36)	1.00 (0.67–1.33)
Internal validation: optimism-adjusted performance	0.78 (0.72–0.85)	−0.02 (−0.44–0.41)	0.77 (0.52–1.07)
External validation: optimism-adjusted performance	0.75 (0.69–0.83)	−0.01 (−0.44–0.40)	0.75 (0.49–1.10)

## Data Availability

The datasets used during the current study are available from the corresponding author on reasonable request.
